# Curcumin - The Nutraceutical With Pleiotropic Effects? Which Cardiometabolic Subjects Might Benefit the Most?

**DOI:** 10.3389/fnut.2022.865497

**Published:** 2022-05-17

**Authors:** Stanisław Surma, Amirhossein Sahebkar, Jakub Urbański, Peter E. Penson, Maciej Banach

**Affiliations:** ^1^Faculty of Medical Sciences in Katowice, Medical University of Silesia, Katowice, Poland; ^2^Club of Young Hypertensiologists, Polish Society of Hypertension, Gdańsk, Poland; ^3^Biotechnology Research Center, Pharmaceutical Technology Institute, Mashhad University of Medical Sciences, Mashhad, Iran; ^4^Neurogenic Inflammation Research Center, Mashhad University of Medical Sciences, Mashhad, Iran; ^5^NomiBiotech Corporation, Złotniki, Poland; ^6^Clinical Pharmacy and Therapeutics Research Group, School of Pharmacy and Biomolecular Sciences, Liverpool John Moores University, Liverpool, United Kingdom; ^7^Liverpool Centre for Cardiovascular Science, Liverpool, United Kingdom; ^8^Department of Preventive Cardiology and Lipidology, Medical University of Lodz, Łódź, Poland; ^9^Cardiovascular Research Centre, University of Zielona Gora, Zielona Góra, Poland; ^10^Department of Cardiology and Adult Congenital Heart Diseases, Polish Mother's Memorial Hospital Research Institute (PMMHRI), Łódź, Poland

**Keywords:** curcumin, cardiovascular risk, cardiovascular disease, treatment, prevention

## Abstract

Despite continuous advances in pharmacotherapy, atherosclerotic cardiovascular disease remains the world's leading killer. Atherosclerosis relates not only to an increased level of cholesterol, but involves the development of atherosclerotic plaques, which are formed as a result of processes including inflammation and oxidative stress. Therefore, in addition to the classical risk factors for ASCVD (such as type 2 diabetes, overweight, obesity, hypertension and metabolic syndrome), residual risk factors such as inflammation and oxidative stress should also be reduced. The most important intervention in ASCVD is prevention, which includes promoting a healthy diet based on products of natural origin. Curcumin, which is often present in the diet, has been demonstrate to confer several benefits to health. It has been shown in numerous clinical trials that curcumin exhibited anti-diabetic, lipid-lowering, antihypertensive, antioxidant and anti-inflammatory effects, as well as promoting weight loss. All this means that curcumin has a comprehensive impact on the most important risk factors of ASCVD and may be a beneficial support in the treatment of these diseases. Recently, it has also been shown that curcumin may have a beneficial effect on the course of SARS-CoV-2 infection and might be helpful in the prevention of long-COVID complications. The aim of this review is to summarize the current knowledge regarding the safety and efficacy of curcumin in the prevention and treatment of cardiometabolic diseases.

## Introduction

The incidence of cardiovascular disease (CVD) in the world in 2019 was 523 million (95% uncertainty interval, UI: 497-550). The number of patients with CVD doubled between 1990 and 2019. The number of CVD deaths steadily increased from 12.1 million (95% UI: 11.4–12.6) in 1990 to 18.6 million (95% UI: 17.1–19.7) in 2019 (1). Of all CVDs, ischemic heart disease (IHD) was the most common cause of death (IHD; 49.2%). CVD causes twice as many deaths as cancers (2).

Such a high prevalence of CVD is a result of the high prevalence in the population of cardiovascular risk factors, such as carbohydrate disorders and diabetes, overweight and obesity, metabolic syndrome, hypertension, and lipid disorders. The worldwide prevalence of prediabetes (measured as impaired glucose tolerance) in 2019 was 7.5%, while it is estimated that by 2045 it will increase to 8.6% (3). The prevalence of diabetes in the world is also increasing. In 1990, the prevalence of this disease was 211.2 million (95% UI: 196.0–228.5), while in 2017 the number increased to 476.0 million (95% UI: 436.6–522.8) (4). The global prevalence of overweight and obesity is also significantly increasing. According to WHO (World Health Organization) Global Health Observatory data, in 2016, the prevalence of overweight and obesity was 1.9 billion and 650 million, respectively (5). It is estimated that in 2030 the number of overweight and obese patients will amount to 2.16 billion and 1.12 billion, respectively (6). The prevalence of the metabolic syndrome is also increasing, especially among young adults (7). Another important risk factor for CVD with an increasing prevalence is hypertension. The number of people diagnosed with hypertension doubled from 1990 to 2019, from 331 (95% CI: 306–359) million women and 317 (95% CI: 292–344) million men in 1990 to 626 (95% CI: 584–668) million women and 652 (95% CI: 604–698) million men in 2019 (8). Moreover, dyslipidemias are extremely common and contribute substantially to the occurrence of atherosclerotic CVD (ASCVD) (9). ASCVD is defined as coronary artery disease (CAD), cerebrovascular disease, or peripheral arterial disease (PAD) of atherosclerotic origin. ASCVD represents the most common cause of morbidity and mortality worldwide (10).

It should be mentioned that the current coronavirus disease 2019 (COVID-19) pandemic has been associated with an increasing burden of CVD risk factors in the population. This may result in future substantial increases in the incidence of CVD (11). Moreover, it is extremely important to control risk factors for CVD and to manage CVD effectively during the pandemic to improve the prognosis of COVID-19 patients (12).

Given the extremely unfavorable epidemiological data on CVD and its risk factors, new and effective therapies are being sought for the condition. Pharmacotherapy and lifestyle modifications remain the cornerstones of the prevention and treatment of CVD. However, additional interventions are needed to improve glycemic parameters, lipid parameters, blood pressure, and residual CVD risk factors, such as oxidative stress and chronic inflammation. The potential exploitation of non-traditional CVD risk reduction methods, such as use of curcumin (a very promising nutraceutical) is an area of growing scientific and clinical interest.

It is also very important to note that the prevalence of ASCVD is greatest in low- and middle-income countries, where access to pharmacological therapies can be limited (13). Moreover, in the United States, it has been observed that less affluent people are most likely to suffer from ASCVD (14). In this light, the availability of a cheap and commonly available food/nutraeutical such as curcumin could have an important role in the prevention of ASCVD.

This review summarizes current safety data for curcumin and its impact of on risk factors for ASCVD such as pre-diabetes, diabetes, obesity, metabolic syndrome, hypertension and hypercholesterolemia. Furthermore, data on the effect of curcumin on residual ASCVD risk factors such as inflammation and oxidative stress are provided.

In a position paper from an International Lipid Expert Panel (ILEP) (Table 1A), and the latest guidelines for the diagnosis and treatment of lipid disorders in Poland (Table 1B) as well as in the ILEP position paper on the anti-inflammatory effects of nutraceuticals (Table 1C) emphasis was made on the important role of curcumin in the prevention of ASCVD.

**Table 1:** Clinical relevance of curcumin based on existing recommendations: **(A)** lipid lowering properties based on the International Lipid Expert Panel (ILEP) position paper (15), **(B)** the place of curcumin in lipid-lowering therapy based on the Polish guidelines (16), **(C)** the role of curcumin in managing inflammatory parameters based on the ILEP position paper (17).

**Class**	**Level**	**Acive daily doses**	**Expected effects on LDL-C**	**Effects on other CV risk biomarkers**	**Direct vascular effects**
**(A)**					
IIa	B	1–3 g	−5%	↓ TG, Lp (a), glucose, HbA_1c_, HOMA index, hs-CRP, TNF-α, IL-6, ↑ adiponectin, HDL-C	↑ FMD, ↓ PWV
**Name**		**Recommended dosage**	**Expected effects on LDL-C**	**Class of recommendation**	**Level of recommendation**
**(B)**				
Curcumin		0.5–3 g	−5 to −10%	IIa	A
**Nutraceuticals**	**Class**	**Level**	**Impact on markers of inflammation**
**(C)**			
Curcumin	IIa	B	A significant decrement in serum concentrations of TNF-α (−4.69 pg/ml), IL-6, TGF-β and MCP-1

*LDL-C, low density lipoprotein cholesterol; CV, cardiovascular; TG, trigliceryde; Lp (a), lipoprotein a; HOMA index, Homeostasis Model Assessment of Insulin Resistance; CRP, C-reactive protein; TNF-α, tumor necrosis factor α; IL-6, interleukin 6; HDL-C, high density lipoprotein cholesterol; FMD, flow-mediated dilation; PWV, pulse wave velocity; TGF-β, transforming growth factor β; MCP-1, monocyte chemoattractant protein-1. The colors are assigned to specific letters (A, B, or C)*.

The use of nutraceuticals, including curcumin, may be useful in a range of clinical situations, including patients with mild to moderately elevated low density lipoprotein cholesterol (LDL-C) concentrations not treated with pharmacotherapy and with a low global risk of ASCVD; patients treated with statins who are unwilling or unable (as in the case of statin intolerance) to increase the dose or intensity of a statin (or add additional LDL-C lowering agents) regardless of the global risk of ASCVD and patients reluctant to take conventional LDL-C lowering pharmacotherapy (18, 19).

Another important justification for the topic under consideration is the fact that curcumin may be beneficial in supporting the treatment of COVID-19 (20), which is currently a challenge for healthcare systems worldwide. A recent systematic review by Vahedian-Azimi et al. showed that curcumin supplementation in patients with COVID-19 led to a significant decrease in common symptoms, duration of hospitalization and all-cause mortality. Moreover, a significant decrease in proinflammatory cytokines such as IL1β and IL6, with a concomitant significant increase in anti-inflammatory cytokines, including IL-10, IL-35 and TGF-α was observed. Thus, curcumin supplementation may offer an efficacious and safe option for improving COVID-19 disease outcomes and may be helpful in the prevention of long-term complications of COVID-19 (21, 22).

### Summary and Take Home Message

Owing to its lipid-lowering and anti-inflammatory properties, curcumin is recommended for the prevention and treatment of ASCVD. Due to the widespread availability of curcumin, it can be an important component of the prevention of ASCVD (and may be used to support treatment) in low–and middle-income countries.

## Curcumin–A Short Overview

**Figure 1:**
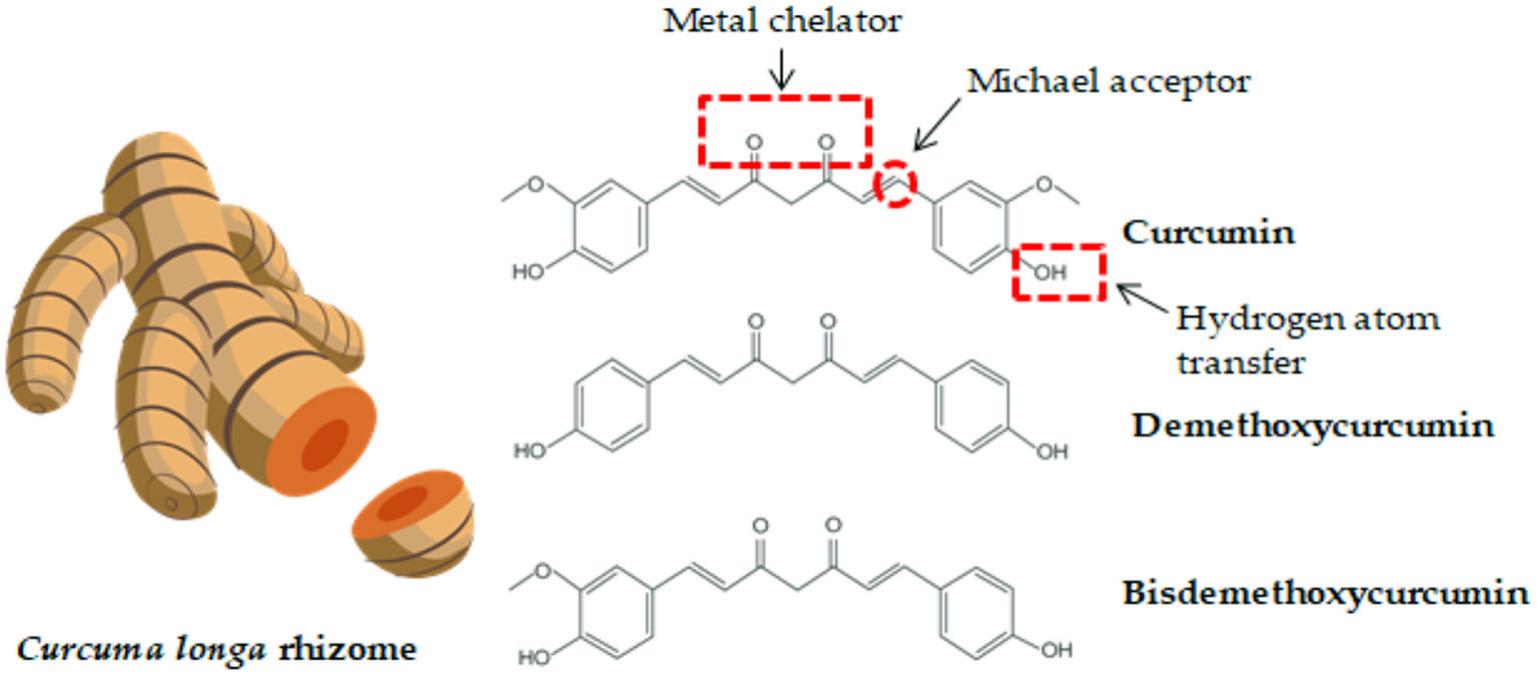
*Curcuma longa* rhizome and the chemical structure of curcumin and its derivatives. Functional groups in curcumin components which contribute to its activity and bioavailability are highlighted (24, 25).

Turmeric (*Curcuma longa*) is a plant related to the ginger family (*Zingiberaceae*), which originated from India and is currently grown in several other parts of the world, including Southeast Asia, China, and Latin America (23). Turmeric consists of ~70% carbohydrates, 13% water, 6% protein, 6% essential oils (phellandrene, sabinene, cineol, borneol, zingiberene, and sesquiterpenes), 5% fat, 3% mineral (potassium, calcium, phosphorus, iron, and sodium), 3–5% curcuminoids, and trace amounts of vitamins (B_1_, B_2_, C, and niacin). Among the curcuminoids, curcumin accounts for approximately 77%, demethoxycurcumin accounts for 17% and bisdemethoxycurcumin accounts for 3–6% (Figure 1) (23, 26, 27).

The healing and health-promoting properties of turmeric were first reported in Middle Eastern medicine, or more precisely in the Indian Ayurverda (23).

The chemical structure of curcumin was described in 1910 by Polish chemists (28). Curcumin [1,7-bis (4-hydroxy-3-methoxyphenyl)-1,6-heptadiene-3,5-dione] is a beta-diketone and consists of two feruloyl residues linked by a carbon atom^1^. The compound owes its characteristic yellow color to the presence of phenolic rings. Curcumin is a phenolic molecule that can exist in two different forms containing the same number of these same atoms in the molecule, but in different inter-convertible isomers (so-called tautomers). Curcumin tautomers exist are in the keto-enol form (23, 26). Phenol groups in the structure of curcumin explain its ability to eliminate oxygen free radicals such as: hydrogen peroxide (H_2_O_2_), superoxide anion (${\text{O}}_{2}^{.-}$) and nitric oxide (NO) (29). Curcumin metabolites are excreted from the body primarily in the feces, and, to a lesser extent, in the urine (29).

Curcumin exhibits a range of pharmacological and biological actions that have been demonstrated in both *in vitro* and *in vivo* studies, and include antioxidant, cardio-protective, anti-inflammatory, anti-microbial, nephro-protective, anti-neoplastic, hepato-protective, immunomodulatory, hypoglycaemic and anti-rheumatic effects (29).

### Summary and Take Home Message

Curcumin is the most common curcuminoid found in turmeric which is widely used in natural medicine.

## Bioavailability of Curcumin

Turmeric (and therefore curcumin) has been used for thousands of years in traditional Ayurvedic medicine and in the medical systems of countries in which this rhizome occurs naturally and forms part of the regular diet. It has been used as an anti-inflammatory drug and as a remedy for a number of diseases and health conditions (29). However, there are no clinical data that clearly confirms its effectiveness in this regard. Taking into account the growing body of research results, it can be concluded with a high degree of certainty that oral administration of curcumin as a dietary component in a naturally occurring form, (contained in both fresh and dried turmeric used as a seasoning and food ingredient, and administration of turmeric in the form of standardized curcuminoid extract from the turmeric rhizome) does not result in reproducible health-enhancing effects, probably because of the low bioavailability of curcumin delivered in this manner (24).

This issue has been discussed in numerous reviews, the general conclusion of which is that the basic obstacle in conducting structured clinical trials, resulting from the chemical structure of the curcumin molecule, is two major and related problems–low water solubility and subsequent low bioavailability (24, 30, 31). Moreover, most of the published studies results refer to the different combinations of various doses of curcumin administered together with substances referred to as “bioavailability enhancers” such as piperine. Piperine can enhance permeation of circumin through the epithelial barrier by inducing changes in membrane dynamics, however, due to its biological activity it might interfere not only with the curcumin molecule, but also exert some additional effects (25). The biological activity of piperine has been reviewed in Gorgani et al. (32). It is also worth mentioning that piperine, in addition to its role in increasing the bioavailability of curcumin, also directly affects ASCVD risk factors. Piperine has been found to have antidiabetic, anti-inflammatory, antioxidant and lipid-lowering effects (33). This means that in some studies where curcumin was used together with piperine, a synergistic effect may have occurred. As a result, curcumin is included in the so-called PAINS (pan-assay interference compounds), i.e., molecules with a structure that makes it impossible to study because of artifacts that prevent elucidation of a clear clinical picture (34).

The chemical structure of curcumin makes this compound practically insoluble in water–with solubility estimated at 3.12 mg/l at 25°C^2^ and insoluble in oil (35). Curcumin is soluble in some organic solvents such as methanol (4.44 mg/mL), 2-butanone (2.17 mg/mL), ethanol (5.6 mg/mL), isopropanol (3.93 mg/mL), acetone (7.75 mg/mL), 1,2-dichloroethane (0.5125 mg/mL) and dimethyl solphooxide (DMSO) (20 mg/mL) (36). Due to its very low solubility in water and fats, curcumin has a very low bioavailability. The absorption of this compound is too low for it to exhibit significant biological effects.

Curcumin is considered to be a non-toxic substance (37). A comprehensive review of the subject has been published by Soleimani et al. It has been clearly demonstrated that taking doses of up to 6 g of curcumin daily in patients with breast cancer (38) or 2 x 500 mg of curcumin with increased bioavailability were safe. In patients with fatty liver disease, curcumin does not cause side effects (39). According to EFSA recommendations, a safe dose of curcumin is up to 3 mg/kg of body weight^3^. It is worth bearing in mind that this dose refers to curcuminoid extract and does not take into account the potentially increased bioavailability of some formulations.

To solve the problem of low bioavailability of native curcumin, without resorting to the use of biologically active compounds such as piperine, in recent years a number of pharmaceutical strategies have been proposed. The approaches include dispersion, nano- and micro- formulations, encapsulation with polysaccharides and lipids, micellization and others. The formulations improve bioavailability by increasing absorption, stabilizing curcumin, or delaying its release.

It has been shown that dispersion of curcuminoids in an aqueous environment significantly improves their bioavailability (40). A common way to obtain curcuminoids dispersible in water is to dissolve them in 70–80% ethanol together with β-cyclodextrin, followed by heating and mixing for at least 4 h at a temperature of 70°C (41, 42). Solvents (ethanol and water) are usually removed by spray drying, vacuum evaporation or freeze drying. This method of obtaining curcuminoids dispersible in water has several disadvantages. Unfavorable chemical transformations of curcuminoids occur in the in the water-ethanol environment as a result of thermal decomposition. Research has demonstrated that in an aqueous environment when temperature is elevated temperature, even at the most favorable pH (curcuminoids are most stable at a pH between 3 and 6) curcuminoids undergo decomposition (43). The preparation of curcuminoids dispersible in water should be carried out at the lowest possible temperatures.

Other formulations of curcuminoids dispersible in water include curcumin adsorbed on silicates, e.g., silicon dioxide (44) or on microcrystalline cellulose with the addition of soy lecithin (45). Several approaches to prepare formulation based on curcuminoid extract suspended in fat particles have been proposed with the addition of phosphatidylcholine (46) or in short chain triacylglycerols with the addition of free fatty acids and polyglycerol esters, stabilized with hydroxypromyl methylcellulose, sodium alginate and microcrystalline cellulose (47) or curcumin powder dispersed in triacetin (glycerol triacetate) and a mixture of lipids obtained from the seeds of soybean, oil palm and rapeseed (48).

Other approaches include 20–28% of turmeric extract (a mixture of three curcuminoids), suspended in a mixture comprised of 63–75% of polyvinylpyrrolidone (E 1201), 10–40% cellulose derivatives with the addition of 1–3% of natural antioxidants (40), or essential oil consisting of 45% ar- turmerone and curcuminoids (49) or curcuminoids in combination with γ-cyclodextrin (50). Nano-colloidal preparation described by Sasaki et al. comprises up to 30% of curcuminoids with glycerin and Ghatti gum (51).

It should be emphasized that the data on the bioavailability of curcumin (curcuminides) in the form of complex formulations described above are inconclusive. Up to date, no comprehensive compilation of the data on bioavailability of a number of leading preparations, made in a uniform experimental system based on the use of a unified method, has been published. This applies to both the experimental model (data are mainly based on animal models, including mice and rats, with a small number of tests in humans), the method of administration, the dose per kilogram of body weight, and the method of determining the level of curcuminoids (taking into account the curcuminoid profile) in plasma, the time points of measurement, including the point where the concentration peak is reached, allowing an approximation of the absorption kinetics. The published review summaries are not based on a uniform baseline, due to the use of standardized but curcuminoid (not curcumin) extracts obtained by various extraction methods, and their very low solubility in water can significantly disturb the actual level of growth bioavailability, therefore the data and claims regarding the bioavailability must be considered as non-conclusive. Studies have demonstrated that the form of curcumin administration and the composition of the food in which it is found can significantly affect absorption (31).

Regardless of the research model and the preparation, curcumin reaches its peak concentration within 45–120 min after oral administration, and its level gradually decreases, returning to the base value. within 6–10 h after consumption of the preparation. Depending on the administered dose and the experimental model and physiological state, the maximum concentration of curcumin in the blood plasma is observed in a very wide range, and can be as high as 3,200 ng/ml (52–54).

### Summary and Take Home Message

Due to its chemical structure, curcumin is characterized by low bioavailability from normal foods. Several methods have been developed to increase the bioavailability of curcumin, including nano-curcumin formulation.

## Curcumin in Patients With Diabetes

The effectiveness of curcumin in the prevention of diabetes and its treatment support has been the subject of several randomized controlled clinical trials (Table 2).

**Table 2:** Summary of studies on the effects of curcumin on the prevention and control of type 2 diabetes.

**References**	**Type of study**	**Sample size**	**Curcumin type and dosage**	**Intervention time**	**Results**	**Safety**	**Conclusions**
Karandish et al. (55)	RCT	84 overweight or obese patients with prediabetes stasus	Curcumin 500 mg/day	3 months	In curcumin group: • FBG: ↓ (*p* < 0.001) • Postprandial glucose level: ↓ (*p* < 0.001) • HbA_1C_: ↓ (*p* < 0.001) • Insulin (μU/ml): ↓ (*p* = 0.006)	No serious adverse events were identified	Curcumin: √ Antidiabetic properties
Mokhtari et al. (56)	RCT	60 patients with diabetic foot ulcer	Nano-curcumin 80 mg/day	12 weeks	• Serum insulin level: ↓ (*p* = 0.01) • Insulin resistance: ↓ (*p* = 0.02) • Insulin sensivity: ↑ (*p* = 0.008) • FBG: ↓ (*p* = 0.02) • TC: ↓ (*p* = 0.001) • LDL-C: ↓ (*p* < 0.001) • TAC: ↑ (*p* < 0.001) • GSH: ↑ (*p* = 0.01) • Wound healing parameters: ↔	Not reported	Curcumin: √ Improving metabolic parameters
Thota et al. (57)	RCT	29 patients with high risk of DMT2 and Alzheimer's disease	Curcumin 1 g/day	12 weeks	• Circulating GSK-3β: ↓(*p* = 0.0068) • IAPP: ↓ (*p* = 0.0163) • HOMA2-IR: ↓ (*p* = 0.0142) • Insulin: ↓ (*p* = 0.0115)	The use of curcumin for 12 weeks (1 g/day) was well tolerated	Curcumin: √ Antidiabetic properities √ ↓ risk of Alzheimer's disease
Shafabakhsh et al. (58)	RCT	60 patients with DMT2 and CHD	Curcumin 1 g/day	12 weeks	• PSQI:↓ (β = −1.27; 95% CI: −2.27 to −0.31; *p* = 0.01) • MDA: ↓ (β = −0.20 μmol/l; 95% CI: −0.36 to −0.04; *p* = 0.01) • TAC: ↑ (β = 75.82 mmol/l; 95% CI: 3.400–148.25; *p* = 0.04) • GSH level: ↑ β = 63.48 μmol/l; 95% CI: 26.58–100.37; *p* = 0.001) • PPAR-γ: upregulated (*p* = 0.01)	Not reported	Curcumin: √ Improving psychological status √ Antioxidant properties √ Anti-inflammatory properties
Asadi et al. (59)	RCT	80 patients with DMT2 and diabetic sensorimotor polyneuropathy	80 mg nano-curcumin/day	8 weeks	• Depression, anxiety, and stress level (DASS-21-items): ↓ (*p* = 0.02) • Mean score of anxiety: ↓ (*p* = 0.009)	Curcumin was safe and well-tolerated in this study	Curcumin: √ Reducing depression and anxiety levels in patients with diabetic polyneuropathy.
Shafabakhsh et al. (60)	RCT	60 patients with diabetes on hemodialysis	80 mg nano-curcumin/day	12 weeks	• FBG: ↓ (β = −19.68 mg/dl, 95% CI: −33.48 to −5.88; *p* < 0.05) • Serum insulin level: ↓ (β = −1.70 μIU/ml, 95% CI: −2.96 to −0.44; *p* < 0.05) • TG: ↓ (β = −16.13 mg/dl, 95% CI: −31.51 to −0.75; *p* < 0.05)	Not reported	Curcumine: √ Beneficial effects on metabolic profile in patients with diabetes on hemodialysis
					• VLDL-C:↓ (β = −3.22 mg/dl, 95% CI: −6.30 to −0.15; *p* < 0.05) • TC: ↓ (β = −17.83 mg/dl, 95% CI: −29.22 to −6.45; *p* < 0.05) • LDL-C: ↓ (β = −15.20 mg/dl, 95% CI: −25.53 to −4.87; *p* < 0.05) • TC/HDL-C ratio: ↓ (β = −1.15, 95% CI: −0.2.10 to −0.21; *p* < 0.05) • hsCRP: ↓ (β = −0.78 mg/l, 95% CI: −1.41 to −0.15; *p* < 0.05) • MDA: ↓ (β = −0.25 μmol/l, 95% CI: −0.45 to −0.04; *p* < 0.05) • TAC: ↑ (β = 52.43 mmol/l; 95% CI: 4.52–100.35; *p* < 0.05) • Total nitrite levels: ↑ (β = 3.62 μmol/l, 95% CI: 2.17–5.08; *p* < 0.001)		
Funamoto et al. (61)	RCT	33 patients with IGT and DMT2	180 mg/day highly absorbable curcumin (Theracurmin®)	6 months	• HbA_1C_: ↔ • TG: ↓ (*p* = 0.015) • In the placebo group, a significant ↑ in blood glucose concentration (*p* = 0.017) and AT-LDL (*p* = 0.024), while in the curcumin group, a non-significant change (*p* = 0.124 and *p* = 0.722)	Not reported	Curcumin: √ Slight improvement in lipid profile
Vanaie et al. (62)	RCT	46 patients with DMT2	Curcumin 500 mg 3x/day	16 weeks	• Proteinuria: ↓ (900.42 ± 621.91 at the baseline to 539.68 ± 375.16 after intervention; *p* = 0.002)	Epigastric pain in one subject	Curcumin: √ Effective adjuvant in therapy for ameliorating macroscopic proteinuria in DMT2 subjects
Adibian et al. (63)	RCT	44 patients with DMT2	1500 mg/day	10 weeks	• TG: ↓ (*p* = 0.4) • hsCRP: ↓ (*p* = 0.008) • FBG: ↓ (*p* = 0.027) • Serum adiponectin level: ↑ (*p* = 0.03) • Body weight: statistically reduced	Not reported	Curcumin: √ Improving lipids parameters √ Anti-inflammatory properties √ Antidiabetic properties
Asadi et al. (64)	RCT	80 patients with DMT2 and diabetic sensorimotor polyneuropathy	80 mg nano-curcumin/day	8 weeks	• FBG: ↓−14.80 (27.78; *p* = 0.004) • HbA_1C_: ↓−0.70 (0.88; *p* < 0.001) • Total score of neuropathy: ↓−2.07 (2.1; *p* < 0.001)	Curcumin was safe and well-tolerated in this study	Curcumin: √ Improved and reduced the severity of diabetic sensorimotor polyneuropathy
					• Total reflex score: ↓−0.65 (1.6; *p* = 0.04)		in patients with DMT2
Srinivasan et al. (65)	RCT	136 patients with DMT2	400 mg of *Curcuma longa* 3x/day	3 months	• Carotid-femoral PWV: ↓ (*p* = 0.002) • Left brachial-ankle PWV: ↓ (*p* = 0.001) • Aortic augmentation pressure: ↓ (*p* = 0.007) • Aortic augmentation index: ↓ (*p* = 0.007) • Aortic augmentation index at heart rate 75: ↓ (*p* = 0.018)	One subject reported increased freuency, and one reffered to upper adbominal pain	Curcumin: √ Decreases arterial stiffness in DMT2 subjects
Thota et al. (66)	RCT	64 patients with high risk of DMT2	1 g/day	12 weeks	• Fasting insulin: ↓ 18,79% (*p* < 0.01) • HOMA2-IR: ↓ (*p* < 0.01) • TG: ↓ (*p* = 0.019) • AIP: ↓ (*p* = 0.025)	Curcumin was well-tolerated by the subjecs and no adverse events was reported	Curcumin: √ Antidiabetic properities √ Improvement of lipid profile
Hodaei et al. (67)	RCT	53 patients with DMT2	500 mg 3x/day	10 weeks	• Weight: ↓ – 0.64 ± 0.22 kg (*p* < 0.05) • BMI: ↓ 0.3 ± 0.03 kg/m2 (*p* < 0.05) • Waist circumference: ↓ – 1.2 ± 0.4 cm (*p* < 0.05) • FBG: ↓−7 ± 2 mg/dl (*p* < 0.05)	The subjects did not report serious side effects	Curcumin: √ Antidiabetic properities √ Improvement weight control
Thota et al. (68)	Cross-over RCT	16 healthy subjects	Curcumin 180 mg/day	4 test days separated by a week	• Postprandial glucose level: ↓ 60.6% (*p* = 0.0007) • AUC for change in blood glucose level: ↓ 36% (*p* = 0.003) • Postprandial insulin (AUC): ↓ 26% (*p* = 0.01)	Not reported	Curcumin: √ Beneficial effect on carbohydrate metabolism–anti-diabetic properties
Panahi et al. (69)	RCT	100 patients with DMT2	Curcuminoids (500 mg/day) + piperine (5 mg/day)	3 months	• Serum glucose level: ↓−9 ± 16 mg/dl (*p* = 0.048) • C-peptide: ↓−0.6 ± 0.8 ng/ml (*p* < 0.001) • HbA_1C_: ↓−0.9 ± 1.1% (*p* < 0.001) • Serum GPT level: ↓−2 ± 6 (*p* = 0.032) • Serum GOT level: ↓−3 ± 5 (*p* = 0.002) • hs-CRP: ↔	No report of any side effects suggesting the safety of the administered combination	Curcuminoids: • Improving glycemic and hepatic parameters
Panahi et al. (70)	RCT	118 patients with DMT2	Curcuminoids 1,000 mg/day co-administered with piperine 10 mg/day	8 weeks	• Weight: ↓−1.0 kg (95% CI: −2.0 to −1.0; *p* < 0.001) • BMI: ↓−0.36 kg/m^2^ (95% CI: −0.70 to −0.32; *p* < 0.001) • Serum TAC: ↑ (*p* < 0.001) • SOD activity: ↑ (*p* < 0.001) • MDA level: ↓ (*p* < 0.001)	Curcuminoids were safe and no severe adverse events were reported during the course of study	Curcuminoids: √ Antioxidant effect
Panahi et al. (71)	RCT	118 patients with DMT2	Curcuminoids 1,000 mg/day co-administered with piperine 10 mg/day	12 weeks	• TC: ↓−21.86 ± 25.78 (*p* = 0.023) • non-HDL-C: −23.42 ± 25.13 (*p* = 0.014) • Lp(a): ↓−1.50 ± 1.61 (*p* = 0.001) • HDL-C: ↑ 1.56 ± 4.25 (*p* = 0.048) • TG: ↔ • LDL-C: ↔	No report of any side effects suggesting the safety of the administered combination	Curcuminoids: √ Reduced risk of cardiovascular events in dyslipidemic patients with DMT2
Panahi et al. (72)	RCT	118 patients with DMT2	Curcuminoids 1,000 mg/day co-administered with piperine 10 mg/day	12 weeks	• Leptin: ↓ (*p* < 0.001) • TNF-α: ↓ (*p* < 0.001) • Leptin:adiponektin ratio: ↓ (*p* < 0.001) • Adipokin: ↑ (*p* = 0.032) • Ghrelin: ↔	No report of any side effects suggesting the safety of the administered combination	Curcuminoids: √ Improvement of the adipokines profile and anti-inflammatory effect
Jiménez-Osorio et al. (73)	RCT	101 patients (50 with non-diabetic proteinuric CKD and 51 subjects with diabetic proteinuric CKD)	Curcumin 320 mg/day	8 weeks	• Proteinuria: ↔ • eGFR: ↔ • Lipid profile: ↔ • Lipid peroxidation: ↓ (non-diabetic proteinuric CKD patients; *p* < 0.05) • Antioxidant capacity: ↑ (diabetic proteinuric CKD; *p* < 0.05)	Not reported	Curcumin: √ Reduce oxidative stress
Rahimi et al. (74)	RCT	80 patients with DMT2	Nano-curcumin 80 mg/day	3 months	• HbA_1C_: ↓ (*p* = 0.02) • FBG: ↓ (*p* = 0.004) • TG: ↓ (*p* = 0.05) • BMI: ↓ (*p* = 0.019)	Not reported	Curcumin: √ Improved metabolic parameters in subjects with DMT2
Yang et al. (75)	CT	14 patients with diabetic kidney disease	Curcumin 500 mg/day	15–30 days	•↓ renal albumin excretion • MDA: ↓ (*p* < 0.001) • LPS: ↓ (*p* < 0.01) • IκB: ↑ (*p* < 0.01) • SOD1 & SOD2: ↑ (*p* = 0.01) • Caspase 3: ↓ (*p* < 0.01) Improves the composition of the gut microbiota: •↑*Bacteroides* •↑*Bifidobacterium* • ↑ *Lactobacillus*	Not reported	Curcumin: √ Reduction of albuminuria, anti-oxidative and anti-inflammatory properties
Na et al. (76)	RCT	100 patients with overweight/ obesity and DMT2	Curcuminoids 300 mg/day	3 months	• A-FABP: ↓ (*p* < 0.001) • CRP: ↓ (*p* < 0.001) • TNF-α: ↓ (*p* = 0.047) • IL-6: ↓ (*p* < 0.001) • SOD activity: ↑ (*p* = 0.005)	Not reported	Curcuminoids: √ Improved metabolic parameters in patients with DMT2
Chuengsamarn et al. (77)	RCT	213 patients with DMT2	Curcuminoids 250 mg 2x/day	6 months	• PWV: ↓ (*p* < 0.001) • Adiponectin: ↓ (*p* < 0.001) • Leptin: ↓ (*p* < 0.001) • HOMA-IR: ↓ (*p* < 0.001) • TG: ↓ (*p* < 0.001) • Uric acid: ↓ (*p* < 0.001) • Visceral fat: ↓ (*p* < 0.05) • Total body fat: ↓ (*p* < 0.001)	6-months curcumin intervention was well-tolerated, with a very few adverse effects	Curcuminoids: √ Antiathero- sclerotic effect
Na et al. (78)	RCT	100 patients with overweight/ obesity and DMT2	Curcuminoids 300 mg/day (placebo = 50 curcumin oids = 50)	3 months	• FBG: ↓ (*p* < 0.01) • HbA_1C_: ↓ (*p* = 0.031) • HOMA-IR: ↓ (*p* < 0.01) • Serum FFAs: ↓ (*p* < 0.01) • TG: ↓ (*p* = 0.018) • LPL activity: ↑ (*p* < 0.01)	Not reported	Curcuminoids: √ Glucose-lowering effect √ Decrease in serum FFAs
Chuengsamarn et al. (79)	RCT	240 prediabetes patients	Curcumin 1,5 g/day	9 months	•↓ risk of progressing to DMT2 (16.4% *vs* 0.0%, *p* < 0.001) • Body weight: ↓ (*p* < 0.05) • WC: ↓ (*p* < 0.05) • FBG: ↓ (*p* < 0.01) • OGTT at 2h: ↓ (*p* < 0.01) • HbA_1C_: ↓ (*p* < 0.01) • Insulin: ↓ (*p* < 0.05) • HOMA-IR: ↓ (*p* < 0.001) • HOMA-β: ↓ (*p* < 0.01) • Adiponectin: ↑ (*p* < 0.05) • C-peptide: ↓ (*p* < 0.05)	The use of curcumin extract for 9 months (1.5 g/day) was not associated with severe side effects	Curcumin: √ Antidiabetic properities √ Improving the function of β cells

*RCT, randomized controlled clinical trial; FBG, fasting blood glucose; HbA_1C_, glycated hemoglobin; TC, total cholesterol; LDL-C, low density lipoprotein cholesterol; TAC, total antioxidant capacity; GSH, glutathione; GSK-3β, glycogen synthase kinase 3 beta; DMT2, diabetes mellitus type 2; IAPP, islet amyloid polypeptide; HOMA-IR, Homeostasis Model Assessment of Insulin Resistance; CHD, coronary heart disease; PSQI, Pittsburgh Sleep Quality Index; MDA, malondialdehyde; PPARγ, peroxisome proliferator- activated receptor gamma; TG, triglyceride; VLDL-C, very-low deinsity lipoprotein cholesterol; hsCRP, high-sensitivity C-reactive protein; IGT, impaired glucose tolerance; AT-LDL, α1-antitrypsin-LDL complex; PWV, pulse wave velocity; AIP, atherogenic index; BMI, body mass index; AUC, Area under the curve; GPT, glutamic pyruvic transferase; GO, glutamic oxoloacetic transaminase; SOD, superoxide dismutase; Lp (a), lipoprotein a; CKD, chronic kidney disease; eGFR, estimated glomerular filtration rate; A-FABP, adipocyte fatty acid-binding protein; TNF-α, tumor necrosis factor α; IL-6, interleukin 6; LPL, lipoprotein lipase; FFA, free fatty acids; WC, waist circumference; OGTT, oral glucose tolerance test. ↔ - no impact, ↓ - decrease, ↑ - increase*.

A meta-analysis by Zhang et al., which included the results of 4 randomized clinical trials (*n* = 453 subjects) assessed the efficacy and safety of curcumin on glycemic control in patients with diabetes mellitus type 2 (DMT2). In the studies which were eligible for inclusion, curcuminoids (doses: 300–1,000 mg) or curcumin (doses: 1,000–1,500 mg) or nanocurcumin (doses: 80 mg) were used for a period of 8–24 weeks. The obtained results differed depending on the origin of the respondents. The use of curcumin was associated with a lower: HOMA-IR (Middle East: MD = −0.60; 95% CI: −0.74 to −0.46 and Asia Pacific: MD = −2.41; 95% CI: −4.44 to −0.39), glycated hemoglobin (HbA_1C_) (MD = −0.70; 95% CI: −0.87 to −0.54), TC, TG, and LDL-C (Asia Pacific only: MD = −23.45; 95% CI: −40.06 to −6.84 and MD = −54.14; 95% CI: −95.71 to −12.57 and MD = −20.85; 95% CI: −28.78 to −12.92), FBG (Asia Pacific subgroup only: −0.57; 95% CI: −0.79 to −0.36). Moreover, curcumin led to a significant increase in adiponectin levels (MD = 0.50; 95% CI: 0.16–0.83). There were no serious side effects. Based on the results of this meta-analysis, researchers concluded that curcumin may assist in improving insulin resistance, glycemic control, and may result in as decrease in TG and TC in patients with DMT2 (80). A very interesting meta-analysis by Huang et al. including the results of 14 randomized clinical trials (*n* = 1,277 subjects) also assessed the anti-diabetic properties of curcuminoids. Only curcumin (compared to curcuminoids and turmeric) was shown to significantly reduce FBG (SMD −0.42; 95% CI: −0.76 to −0.07, *p* = 0.049). Moreover, the beneficial effect of curcuminoids was dose-dependent–only the use of ≥300 mg/day led to a significant reduction in FBG (SMD −0.58; 95% CI: −1.04 to −0.11, *p* = 0.000). The duration of curcuminoid therapy was also very important - only their use for ≥12 weeks led to a significant reduction in FBG (SMD −0.55; 95% CI: −0.97 to −0.13, *p* = 0.000). The observed effects of curcuminoids on FBG were significant in people with diabetes but not with metabolic syndrome. This meta-analysis also assessed the effect of curcuminoids on HbA_1C_ levels. In the case of HbA_1C_, only the use of curcuminoids (*vs* curcumin) led to a significant decrease in it (SMD −0.414; 95% CI: −0.665 to −0.164, *p* = 0.001). Only the use of curcuminoids in a dose of ≥300 mg / day led to a significant decrease in HbA_1C_ (SMD −0.322; 95% CI: −0.588 to −0.056, *p* = 0.018). Only the duration of the intervention for ≥12 weeks led to a significant reduction in HbA_1C_ (SMD −0.488; 95% CI: −0.790 to −0.185, p = 0.002) (81). Similar results were obtained by de Melo et al. in a meta-analysis including the results of 11 randomized clinical trials. These researchers found that the use of curcuminoids or curcumin led to a decrease in FBG (MD −8.88 mg/dL; 95% CI: −5.04 to −2.72, *p* = 0.005), with isolated curcumin being more potent than curcuminoids (MD −16.02; 95% CI: −29.57 to −2.48 vs. MD = −8.82; 95% CI: −17.30 to −0.34) and this beneficial effect was significant only in people with pre-diabetes or DMT2. Moreover, use of curcumin led to a decrease in the concentrations of HbA_1C_ (MD −0.54%; 95% CI: −1.09 to −0.002, *p* = 0.049) (82). A meta-analysis by Altobelli et al. evaluated the efficacy of curcumin in patients with uncomplicated DMT2. The meta-analysis included the results of 7 randomized clinical trials (*n* = 552 subjects). Curcumin use was been shown to be associated with reductions in: HbA_1C_ (effect size: ES −0.42; 95% CI: −0.72 to −0.11, *p* = 0.008), HOMA-IR (ES −0.41; 95% CI: −0.66 to −0.22, *p* < 0.001), LDL-C (ES −0.28; 95% CI: −0.5 to −0.04, *p* = 0.021), TG (ES −0.57; 95% CI: −0.83 to −0.31, *p* < 0.001), TC (ES −0.30; 95% CI: −0.53 to −0.07, *p* = 0.01). The researchers concluded that the use of daily supplements of curcumin could improve some metabolic parameters in individuals with uncomplicated DMT2 (83).

A systematic review of the literature by Marton et al. of 16 studies found that the antidiabetic properties of curcumin are due to: (1) suppression of oxidative stress and inflammatory process; (2) reduction of fasting blood glucose, glycated hemoglobin, and body mass index; and (3) reduction in triglycerides, very-low density lipoprotein cholesterol (VLDL-C), TC, LDL-C, HDL-C, serum CRP, and plasma malonaldehyde (MDA) (84). Another systematic review of 11 studies (*n* = 1,131 participants) conducted by Mahdavi et al. assessed the efficacy of curcumin in supporting the treatment of type 2 diabetes. It was found that curcumin supplementation led to a reduction in FBS and HbA_1C_. Curcumin also tended to lower HOMA-IR. Patients who had used curcumin for ≥ 12 weeks showed a significant reduction in glycemic indices. Thus, as the authors of this review point out, curcumin may be a promising addition to therapies used to manage DMT2 (85). Another systematic review in which the anti-diabetic properties of curcumin was assessed was conducted by Pivari et al. who summarized the results of numerous *in vitro* and *in vivo* studies. They found strong evidence suggestive of the efficacy of curcumin in DMT2. It has been shown in clinical trials that curcumin is particularly effective in lowering HbA_1C_. The authors of this systematic review also concluded that curcumin has poor bioavailability (86).

In a new review of the literature by Mohammadi et al. the effect of curcumin on glucagon-like peptide-1 (GLP-1), dipeptidyl peptidase-4 (DPP-4), glucose transporters, α-glycosidase, α-amylase, and peroxisome proliferator -activated gamma receptor (PPARγ) were investigated in addition to the standard biomarkers of diabetes described above. These are novel signaling pathways involved in the potential beneficial effects of curcumin for the treatment of diabetes (87). A potential explanation for the effects of curcumin on signaling pathways may be the recently postulated action of curcumin and its structural derivatives, such as C0818 [3,5-(E)-bis(3-methoxy-4-hydroxybenzal)-4-piperidinonehydrochloride], as inhibitors of Hsp90 heat shock protein (88). This is consistent with the results on the role of curcumin in the inhibition of adenovirus replication by disruption of E1A protein *via* Hsp90 dependant pathway (89, 90) and the results on the role of curcumin rescuing the nuclear localization and transactivation activity of mutated PHOX2B carrying the largest expansion of polyAla in CCHS, where curcumin exerts and effect analogous to canonical Hsp90 inhibitors such as 17-AAG (91). Given the role of the Hsp90 protein, the use of curcumin as a low-toxic inhibitor of Hsp90 may be of great importance not only in diabetes, but also in CVD, and this topic certainly deserves further in-depth research.

### Summary and Take Home Message

The use of curcumin in patients at high risk of diabetes and in patients with existing diabetes (with and without complications) is characterized by hypoglycemic, lipid-lowering, anti-inflammatory and antioxidant effects. This is likely to translate into a reduction in cardiovascular risk in this group of patients.

## Curcumin in Patients With Obesity/Metabolic Syndrome

The effectiveness of curcumin in the prevention of diabetes and its treatment support has been the subject of several randomized controlled clinical trials (Table 3).

**Table 3:** Summary of studies on the effects of curcumin on the obesity, metabolic syndrome, and obesity-related diseases.

**References**	**Type of study**	**Sample size**	**Curcumin type and dosage**	**Intervention time**	**Results**	**Safety**	**Conclusions**
Alidadi et al. (92)	RCT	66 subjects with metabolic syndrome	Curcumin 500 mg/day (placebo = 23 curcumin = 23)	12 weeks	Body weight: ↓ PWV: ↓ (*p* = 0.011)	Not reported	Curcumin: Improvement of arterial stiffness and weight
Bateni et al. (93)	RCT	50 subjects with metabolic syndrome	Nano-curcumin 80 mg/day (placebo = 25 curcumin = 25)	12 weeks	TG: ↓ (*p* < 0.05) HOMA-β: ↓ (*p* < 0.05)	Not reported	Curcumin: Improvement of metabolic parameters
Mirhafez et al. (94)	RCT	80 subjects with NAFLD	Curcumin phytosome 250 mg/day (placebo = 40 curcumin = 40)	2 months	Grade of hepatic steatosis: ↓ (*p* = 0.015) AST: ↓ (*p* = 0.007)	No serious adverse events were identified	Curcumin: Hepatoprotective effect in subjects with NAFLD
Jamilian et al. (95)	RCT	60 subjects with PCOS	Curcumin 500 mg/day (placebo = 30 curcumin = 30)	3 months	Weight: ↓ (*p* = 0.03) BMI: ↓ (*p* = 0.03) FBG: ↓ (*p* = 0.002) Serum insulin: ↓ (*p* = 0.02) Insulin resistance: ↓ (*p* = 0.02) Insulin sensivity: ↑ (*p* = 0.02) TC: ↓ (*p* = 0.001) LDL-C: ↓ (*p* = 0.001) TC/HDL-C ratio: ↓ (*p* < 0.001) HDL-C: ↑ (*p* = 0.01) PPAR-γ: upregulated (*p* = 0.03) LDL-R: upregulated (*p* < 0.001)	No serious adverse events were identified	Curcumin: Antidiabetic properties
Sohaei et al. (96)	RCT	51 overweight/ pbese subjects with PCOS	Curcumin 500 mf 2x/day (placebo = 24 curcumin = 27)	6 weeks	Serum insulin: ↓ (*p* = 0.020) QUICKI: ↑ (*p* = 0.003)	Not reported	Curcumin: Antidiabetic properties
Jazayeri-Tehrani et al. (97)	RCT	84 overweight/ obese subjects with NAFLD	Nano-curcumin 40 mg/day (placebo = 42 curcumin = 42)	3 months	HDL-C: ↑ (*p* < 0.05) QUICKI: ↑ (*p* < 0.05) Nefastin: ↑ (*p* < 0.05) Fatty liver degree: ↓ (*p* < 0.05) TNF-α: ↓ (*p* < 0.05) hsCRP: ↓ (*p* < 0.05) IL-6: ↓ (*p* < 0.05) Liver transaminases: ↓ (*p* < 0.05) WC: ↓ (*p* < 0.05) FBG: ↓ (*p* < 0.05) Fasting blood insulin: ↓ (*p* < 0.05) HbA_1C_: ↓ (*p* < 0.05) TG: ↓ (*p* < 0.05) TC: ↓ (*p* < 0.05) LDL-C: ↓ (*p* < 0.05) HOMA-IR: ↓ (*p* < 0.05)	The patients reported no side-effects and side-events associated with treatment during the study	Curcumin: Improvements in inflammation, lipids, and glucose profile
Saraf-Bank et al. (98)	RCT	60 overweight/ obese adolescent girl	Curcumin 500 mg/day (placebo = 30 curcumin = 30)	10 weeks	BMI: ↓ (*p* = 0.019) WC: ↓ (*p* = 0.008) Hip circumference: ↓ (*p* = 0.030) HDL-C: ↑ (*p* = 0.042) TG/HDL-C ratio: ↓ (*p* = 0.021)	Not reported	Curcumin: Slight weight loss diet might have beneficial effects on some cardiovascular risk factors
Saraf-Bank et al. (99)	RCT	60 overweight/ obese adolescent girl	Curcumin 500 mg/day (placebo = 30 curcumin = 30)	10 weeks	IL-6: ↓ (*p* < 0.001) hsCRP: ↓ (*p* = 0.039) TAC: ↑ (*p* = 0.014)	Curcumin was safe nutraceutical	Curcumin: Improve inflammation and oxidative stress
Campbell et al. (100)	RCT	22 obese men	Enhanced bioavailable curcumin 500 mg/day	12 weeks (placebo = 11 curcumin = 11)	Homocysteine: ↓ (p = 0.04) HDL-C: ↑ (*p* = 0.04)	No adverse events were reported in the intervention	Curcumin: Improve homocysteine and high-density lipoprotein concentrations, which may promote favorable cardiovascular health in young
Saadati et al. (101)	RCT	50 subjects with NAFLD	Curcumin 1500 mg/day (placebo = 23 curcumin = 27)	12 weeks	Hepatic fibrosis: ↓ (p < 0.05) TC: ↓ (*p* < 0.05) Serum glucose: ↓ (*p* < 0.05) ALT: ↓ (*p* < 0.05)	Not reported	Curcumin: Improvement of hepatic and metabolic parameters
Mirhafez et al. (102)	RCT	61 subjects with NAFLD	Phospholipidated curcumin 250 mg/day (placebo = 29 curcumin = 32)	8 weeks	HDL-C: ↑ (*p* = 0.01) Adiponectin: ↑ (*p* < 0.001) Leptin: ↓ (*p* < 0.001) Leptin: adiponectin: ↓ (*p* < 0.001)	The curcumin was found to be safe and no patients reported side effects with its use	Curcumin: Improvement of metabolic parameters
Panahi et al. (103)	RCT	87 subjects with NAFLD	Phytosomal curcumin 500 mg 2x/day (placebo = 43 curcumin = 44)	8 weeks	BMI: ↓ (*p* = 0.003) WC: ↓ (*p* = 0.024) ALT: ↓ (*p* < 0.001) AST: ↓ (*p* < 0.001) Hepatic vein flow velocity: ↑ (*p* < 0.001) Portal vein diameter: ↓ (*p* < 0.001) Liver size: ↓ (*p* < 0.001) NAFLD severity: ↓ (*p* < 0.001)	Curcumin was safe and well-tolerated during the course of trial	Curcumin: Improvement of liver parameters
Campbell et al. (104)	RCT	22 obese men	Enhanced bioavailable curcumin 500 mg/day	12 weeks	Aortic stiffness was reduced by curcumin in subjects with increased baseline values	No adverse side effects were reported in the curcumin or placebo groups	Curcumin: might reduce the stiffness in arteries in young, obese men with greater aortic stiffness
Panahi et al. (105)	RCT	100 subjects (intervention group: mean BMI = 25.46 ± 2.46 kg/m^2^	Curcumin 1 g/day (placebo = 50 curcumin = 50)	8 weeks	Adiponectin: ↓ (*p* < 0.001) Leptin: ↓ (*p* < 0.001) Leptin: adiponectin: ↓ (*p* < 0.001)	Curcumin was well-tolerated during the study	Curcumin: Improvement of the adipokine profile
Panahi et al. (106)	RCT	117 subjects with metabolic syndrome	Curcumin 1 g/day (placebo = 58 curcumin = 59)	8 weeks	TNF-α: ↓ (*p* < 0.001) IL-6: ↓ (*p* < 0.001) TGF-β: ↓ (*p* < 0.001) MCP-1: ↓ (*p* < 0.001)	Curcumin was safe and well-tolerated	Curcumin: Decreases serum concentrations of pro-inflammatory cytokines
Esmaily et al. (107)	RCT	30 obese subjects	Curcumin 1 g/day (placebo = 15 curcumin = 15)	1 month	Beck Anxiety Inventory: ↓ (*p* = 0.03)	Not reported	Curcumin: Potential anti-anxiety effect in individuals with obesity
Pierro et al. (108)	RCT	44 overweight subjects with metabolic syndrome	Curcumin 800 mg/day (curcumin = 22 phosphatydylocholine = 22)	1 month	Body weight: ↓ (*p* < 0.01) Body fat: (*p* < 0.01) Hip circumference: (*p* < 0.01) BMI: (*p* < 0.01)	Overall, the therapy was well-tolerated	Curcumine: Positively influence on weight management
Genjali et al. (109)	RCT	30 obese subjects	Curcumin 1 g/day (placebo = 15 curcumin = 15)	1 month	IL-1β: ↓ (*p* = 0.042) IL-4: (*p* = 0.008) VEGF: (*p* = 0.01)	Not reported	Curcumin: Immunomodulatory effects

*RCT, randomized controlled trials; PWV, pulse wave velocity; TG, triglyceride; HOMA, Homeostatic Model Assessment for Insulin Resistance; NAFLD, non-alcoholic fatty liver disease; AS, aspartate transaminase; PCOS, polycystic ovary syndrome; BMI, body mass inex; FBG, fasting blood glucose; TC, total cholesterol; LDL-C, low density lipoprotein cholesterol; HDL-C, high density lipoprotein cholesterol; PPARγ, peroxisome proliferator- activated receptor gamma; LDL-R, low density lipoprotein receptor; QUICKI, Quantitative Insulin Sensitivity Check Index; TNF-α, tumor necrosis factor α; hsCRP, high sensitivity C-reactive protein; IL-6, interleukin 6; WC, waist circumference; HbA1C, glycated hemoglobin; TAC, total antioxidant capacity; ALT, alanine transaminase; TGF-β, transforming growth factor beta; MCP-1, monocyte chemoattractant protein-;1 IL-1β, interleukin 1β; IL-4, interleukin 4; VEGF, vascular endothelial growth factor. ↓ - decrease, ↑ - increase*.

The beneficial properties of curcumin have also been confirmed in numerous meta-analyzes and systematic reviews. In a meta-analysis of the results of 4 studies conducted by Atkin et al., the effect of curcuminoid supplementation on the concentration of leptin was assessed. The duration of the intervention ranged from 4 weeks to 6 months, and the dose of curcuminoids was 250–1,000 mg/day. It was shown that curcuminoids led to a significant decrease in leptin concentration (SMD = −0.695; 95% CI: −1.162 to −0.229, *p* = 0.003) (110). Furthermore, a meta-analysis of 6 randomized clinical trials conducted by Clark et al. found that curcumin supplementation (duration of intervention: 6–39 weeks, dose 200–1,500 mg/day curcumin/curcuminoids) led to a significant increase in adiponectin concentration (WMD: 0.82; 95% CI: 0.33–1.30, *p* < 0.001) (111). A meta-analysis by Azhdari et al., including the results of 7 randomized clinical trials, assessed the efficacy of curcumin in people with metabolic syndrome. The duration of the intervention ranged from 6 to 12 weeks, while curcumin or curcuminoids were used at a dose of 800–2,400 mg/day (up to 20 mg curcumin/kg body weight). Curcumin use was associated with a significant decrease in FBG (WMD = −9.18; 95% CI: −16.70 to −1.66, *p* = 0.000), TG (WMD = −33.65; 95% CI: −51.27 to −16.03, *p* < 0.001) and a significant increase in HDL -C (WMD = 4.31; 95% CI: 1.50–7.11, *p* < 0.001) (112). The anti-inflammatory efficacy of curcumin in subjects with metabolic syndrome was assessed in a meta-analysis conducted by Panahi et al., which included the results of 8 randomized clinical trials. The duration of supplementation in the included studied ranged between 2 and 12 weeks and the administered doses of curcuminoids ranged between 80 mg/day and 6 g/day. It was found that curcumin significantly decreased CRP concentration (WMD = −2.20 mg/l; 95% CI: −3.96 to −0.44, *p* = 0.01) (113). The beneficial effect of curcumin was also confirmed in a meta-analysis conducted by Baziar and Parohan. This meta-analysis assessed the effectiveness of curcumin in patients with non-alcoholic fatty liver disease (NAFLD), which is often a complication of obesity. The meta-analysis included 8 randomized clinical trials with a total of 520 participants. The dose of the supplement ranged from 70 to 3,000 mg/day and the intervention was given for between 8 and 12 weeks. It was found that curcumin led to a significant decrease in BMI (WMD = −0.34 kg/m^2^; 95% CI: −0.64 to −0.04, *p* < 0.05) and waist circumference (WMD = −2.12 cm; 95% CI: −3.26 to −0.98, *p* < 0.001) (114). A very interesting meta-analysis of the results of 11 randomized clinical trials conducted by Mousavi et al. assessed the effect of curcumin on body weight. These studies used curcumin or nanocurcumin or curcuminoids at a dose of 80–1,900 mg/day for 4–13 weeks. A significant effect of curcumin administration on body weight was demonstrated (WMD = −1.14 kg; 95% CI: −2.16 to −0.12, *p* = 0.02) and BMI (WMD = −0.48 kg/m^2^; 95% CI: −0.78 to −0.17, *p* = 0.002). Furthermore, the effect of curcumin on WC was significant in studies that prescribed ≥1,000 mg/day curcumin (*p* ≤ 0.001), those with the intervention duration of ≥ 8 weeks (*p* ≤ 0.001), and those that was performed on overweight subjects (*p* ≤ 0.001) (115). A meta-analysis of the results of 26 randomized clinical trials conducted by Tabrizi et al. summarized the effect of curcumin supplementation on glycemic control and lipid profile in patients with metabolic syndrome. Curcumin supplementation was shown to lead to reduced fasting glucose levels (SMD = −0.78; 95% CI: −1.20, −0.37, *p* < 0.001), HOMA-IR (SMD = −0.91; 95% CI: −1.52, −0.31, *p* = 0.003) and HbA_1C_ (SMD = −0.92; 95% CI: −1.37, −0.47, *p* < 0.001). Moreover, curcumin supplementation was significantly associated with reduced triglyceride concentration (SMD = −1.21; 95% CI: −1.78, −0.65, *p* < 0.001) and total cholesterol reduction (SMD = −0.73; 95% CI: −1.32, −0.13, *p* = 0.01) (116).

A recent systematic review of the results of 28 randomized clinical trials conducted by Safari et al. summarized the knowledge about the effectiveness of curcumin in overweight or obese people. The authors indicate that available studies indicate that curcumin has beneficial impacts on various anthropometric indices in these group of patients (117).

From a clinical point of view, it is important to note that curcumin improves the endocrine function of adipose tissue. A meta-analysis of the results of 6 clinical trials conducted by Simental-Mendía et al. showed that curcuminoid supplementation led to a significant increase in adiponectin levels (WMD = 6.47 ng/mL, 95% CI: 1.85–11.10, *p* = 0.010) (118).

### Summary and Take Home Message

The use of curcumin in patients with overweight/obesity, metabolic syndrome, PCOS, who are objectively characterized by an increased cardiovascular risk, led to the improvement of a number of metabolic parameters affecting this risk.

## Anti-Hypertensive, Lipid-Lowering, Anti-Inflammatory, and Antioxidant Properties of Curcumin

Curcumin has well-documented antihypertensive properties. In a meta-analysis by Hadi et al. including the results of 11 clinical trials, the antihypertensive properties of curcumin were assessed. The duration of intervention was different among studies and ranged between 6 and 24 weeks. The dose of curcumin/turmeric administration ranged from 150 to 2,400 mg/day. A significant reduction in SBP (ES = −1.24 mmHg; 95% CI: −2.26 to −0.22) was demonstrated in studies with ≥12-week curcumin supplementation (119). Interestingly, in an experimental study by Lee et al. it was shown that co-administration of amlodipine and curcumin had a stronger vasorelaxant effect than amlodipine alone. Thus, the use of curcumin and amlodipine may be an effective antihypertensive combination (120).

Curcumin is characterized by lipid-lowering properties, as reflected in the ILEP position paper from (Table 1A) (15), and in the latest guidelines for the diagnosis and treatment of lipid disorders in Poland (Table 1B) (16). A meta-analysis of the results of 20 randomized clinical trials conducted by Simental-Mendía et al. showed that curcuminoid supplementation led to a significant reduction in plasma triglycerides (WMD = −21.36 mg/dL; 95% CI: −32.18, −10.53, *p* < 0.001), and an elevation in plasma HDL-C levels (WMD = 1.42 mg/dL; 95% CI: 0.03–2.81, *p* = 0.046). Importantly, the effects of curcuminoids on lipids were not found to be dependent on the duration of supplementation (121). Similar results were obtained by Yuan et al. in a meta-analysis of the results of 12 randomized clinical trials involving adults with metabolic diseases. Turmeric and curcuminoids supplementation was found to be associated with a reduction in: TG by −19.1 mg/dL (95% CI: −31.7, −6.46 mg/dL, *p* = 0.003), TC by −11.4 mg/dL (95% CI: −17.1, −5.74 mg/dL, *p* < 0.0001), and LDL cholesterol by −9.83 mg/dL (95% CI: −15.9, −3.74 mg/dL, *p* = 0.002). HDL-C was increased by 1.9 mg/dL (95% CI: 0.31–3.49 mg/dL, *p* = 0.02). The beneficial effect of turmeric and curcuminoids supplementation depended on the intervention time (more than 8 weeks) and dose (higher doses) (122). A meta-analysis of the results of 7 randomized clinical trials conducted by Qin et al. also demonstrated lipid-lowering properties of turmeric and curcumin. It was found that turmeric and curcumin significantly reduced serum LDL-C (SMD = −0.340; 95% CI: −0.530, −0.150, *p* < 0.0001) and TG (SMD = −0.214; 95% CI: −0.369, −0.059, *p* = 0.007) (123). In a randomized study by Ferguson et al. involving 70 hypercholesterolaemic individuals, a significant lipid-lowering effect of curcumin was demonstrated, especially when combined with phytosterols (124).

In the ILEP position paper on the anti-inflammatory effects of nutraceuticals (Table 1C), the very favorable effects of curcumin in this area were discussed (17). A meta-analysis of the results of 9 randomized clinical trials conducted by Derosa et al. showed that curcuminoid supplementation significantly reduced circulating IL-6 concentrations (WMD = −0.60pg/mL; 95% CI: −1.06, −0.14, *p* = 0.011). The observed effect was independent of the dose or duration of intervention (125). Moreover, a meta-analysis of the results of 32 randomized clinical trials conducted by Gorabi et al. showed that curcumin supplementation significantly decreased serum levels of IL-1 (WMD = −2.33 pg/mL; 95% CI: −3.33, −1.34, *p* < 0.001) and TNF-α (WMD = −1.61 pg/mL; 95% CI: −2.72, −0.51, *p* < 0.001) compared to the placebo group following treatment (126). A meta-analysis of the results of 32 randomized clinical trials conducted by Ferguson et al. found that curcumin supplementation led to reduction in CRP (WMD = −1.55 mg/L; 95% CI: −1.81, −1.30), IL-6 (WMD = −1.69 pg/mL; 95% CI: −2.56, −0.82), TNF-α (WMD = −3.13 pg/mL; 95% CI: −4.62, −1.64), IL-8 (WMD = −0.54 pg/mL; 95% CI: −0.82, −0.28), MCP-1 (WMD = −2.48 pg/mL; 95% CI: −3.96, −1.00). An increase in concentrations of the anti-inflammatory cytokine IL-10 (WMD = 0.49 pg/mL; 95% CI: 0.10–0.88) was observed (127). The beneficial effect of curcumin supplementation on the reduction of inflammation was confirmed in a meta-analysis of the results of 23 randomized clinical trials by Gorabi et al. It was found that curcumin significantly reduced the level of CRP compared to placebo (WMD = −3.67 mg/L; 95% CI: −6.96, −0.38, *p* = 0.02). The reduction in CRP levels was greatest when a dose of ≤1,000 mg/day was used for > 10 weeks (128). In an interesting meta-analysis of the results of 15 randomized clinical trials conducted by Tabrizi et al. the antioxidant activity of curcumin was assessed in addition to its anti-inflammatory effects. It was demonstrated that the use of supplements containing curcumin resulted in a decrease in IL-6 levels (SMD = −2.08; 95% CI: −3.90, −0.25, *p* = 0.02), hs-CRP (SMD = −0.65; 95% CI: −1.20, −0.10, *p* = 0.02), and malondialdehyde (MDA) concentrations (SMD = −3.14; 95% CI: −4.76, −1.53, *p* < 0.001) (129). The antioxidant properties of curcumin were confirmed by Qin et al. in a meta-analysis of the results of 8 randomized clinical trials. These researchers showed that curcumin supplementation led to reduction in circulating MDA concentrations (SMD = −0.769; 95% CI: −1.059, −0.478) and a significant increase in SOD activity (SMD = 1.084; 95% CI: 0.487–1.680). The antioxidant effect was most pronounced when using curcumin at a dose ≥ 600 mg/day (130). Interestingly, as demonstrated by Panahi et al. supplementation of curcumin (1 g/day for 8 weeks) led to a decrease in uric acid levels (*p* < 0.001), which is also a significant risk factor for CVD (131).

### Summary and Take Home Message

Curcumin use is characterized by general antihypertensive, lipid-lowering, anti-inflammatory and antioxidant effects, which may be used in reducing cardiovascular risk in patients with these risk factors.

## Curcumin as a Support in the Treatment of ASCVD

It has been demonstrated that curcumin is a natural product with anti-atherosclerotic properties (132). The pleiotropic effects of curcumin described above have a very beneficial effect as a support for the treatment of patients with ASCVD. In a study by Mirzabeigi et al. Thirty-three patients with CAD received curcumin (500 mg/day for 8 weeks) or placebo. Curcumin was shown to significantly reduce triglycerides (*p* = 0.01), LDL cholesterol (*p* = 0.03) and VLDL cholesterol (*p* = 0.04) (133). Curcumin is also effective in patients with ASCVD. In a study by Alwi et al. involving 75 patients with acute coronary syndrome, it was shown that the administration of curcumin led to a reduction in total cholesterol level and LDL cholesterol level in these patients (134). In a study by Wongcharoen et al. which included 121 patients after coronary bypass surgery, the effect of curcuminoid supplementation (4 g/day started 3 days before the scheduled surgery and continued until 5 days after surgery) on the risk of MI was assessed. It was shown that incidence of in-hospital MI was decreased from 30.0% in the placebo group to 13.1% in the curcuminoid group (aHR = 0.35; 95% CI: 0.13–0.95, *p* = 0.038). Moreover, a reduction in the level of CRP, MDA, and N-terminal pro-B-type natriuretic peptide was found (135). Curcumin is also effective in controlling the lipid and glycaemic profiles in patients with acute MI. In a randomized clinical trial by Tabaee et al. involving 72 patients with acute MI, curcumin supplementation (500 mg/day, 95% curcuminoids for 8 weeks) led to a reduction in LDL levels (−10.3 ± 20.7 *vs*. +0.2 ± 22.5, *p* = 0.039), an increase in HDL level (+4.5 ± 8.9 *vs*. −1.6 ± 7.7, *p* = 0.002) and a decrease in HbA_1C_ (-0.3 ± 2.2 *vs*. + 1.1 ± 1.3, *p* = 0.002) (136). From a clinical point of view, it is relevant that curcumin can reduce muscle pain and limit muscle damage associated with statin use, making curcumin a very important supplement in patients with statin intolerance (137). An important group of patients at high risk of ASCVD are hemodialysis patients with ESRD. In a study by Afshar et al. which included 54 hemodialysis patients, the effect of curcumin supplementation (nano-curcumin at a dose of 120 mg over 12 weeks) or placebo on inflammation and biomarkers of atherosclerosis was analyzed. A significant reduction in the level of hs-CRP (*p* < 0.001), VCAM-1 (*p* < 0.001) and ICAM-1 (*p* < 0.05) has been demonstrated, which means that curcumin may reduce the inflammation and progression of atherosclerosis in these patients (138). Another group of patients who may benefit from curcumin supplementation are those after PCI. In a study by Silalahi et al., 50 patients with stable CAD after PCI were supplemented with curcumin (5 mg/day was given 7 days prior to PCI until 2 days after PCI) or placebo. It was shown that curcumin significantly reduce the serum hsCRP (*p* = 0.006) and sCD40L (*p* = 0.002) 7 days before PCI to 48 h post-PCI. The decrement of hsCRP (−14.2 *vs*. −7.4%) and sCD40L (−24.3 *vs*. −13.2%) from 24 to 48 h post-PCI was higher in the curcumin group than placebo group. Thus, curcumin supplementation may be effective in reducing inflammation in patients with stable CAD after PCI (139).

### Summary and Take Home Message

Curcumin has a several beneficial effects relevant to the supportive management of patients with ASCVD.

## Conclusions and Perspectives

The impressive efficiency of curcumin in the prevention of ASCVD is highlighted by a recent recent meta-analysis conducted by Ashtary-Larky et al. (140), which included the results of 9 randomized clinical trials (*n* = 510 participants). In the analyzed studies, nano-curcumin was used at a dose of 40–120 mg/day for a period of 6–12 weeks (140). The results of this important meta-analysis are summarized in Table 4.

**Table 4:** The effect of nano-curcumin supplementation on the control of cardiovascular parameters. Based on (140).

**Metabolic parameter**	**WMD**	**95% CI**	**Comments**
TG [mg/dl]	−24.87	−37.34 to −12.40; *p* < 0.001	Baseline TG ≥ 150 mg/dl
	−27.23	−43.11 to −11.35; *p* = 0.001	Obese (>30 kg/m^2^)
TC [mg/dl]	−10.90	−16.40 to −5.39; *p* < 0.001	Baseline TC ≥ 200 mg/dl and obese (>30 kg/m^2^)
LDL-C [mg/dl]	−13.70	−19.26 to −8.13; *p* < 0.001	Baseline LDL-C ≥ 100 mg/dl and obese (>30 kg/m^2^)
HDL-C [mg/dl]	5.77	2.90–8.64; *p* < 0.001	Overall effect
FBG [md/dl]	−18.14	−29.31 to −6.97, *p* = 0.001	Overall effect
Fasting insulin	−1.21	−1.43 to −1.00; *p* < 0.001	Overall effect
HOMA-IR	−0.28	−0.33 to −0.23; *p* < 0.001	Overall effect
SBP [mmHg]	−7.09	−12.98 to −1.20; *p* < 0.001	Overall effect
CRP [mg/l]	−1.29	−2.15 to −0.44; *p* = 0.003	Overall effect
IL-6	−2.78	−3.76 to −1.79, *p* < 0.001	Overall effect

*WM, weighted mean difference; TG, trigliceryde; TC, total cholesterol; LDL-C, low density lipoprotein cholesterol; HDL-C, high density lipoprotein cholesterol; FBG, fasting blood glucose; HOMA-IR, Homeostasis Model Assessment of Insulin Resistance; SBP, systolic blood pressure; CRP, C-reactive protein; IL-6, interleukin 6*.

Thus, in this meta-analysis, it was found that nano-curcumin supplementation was associated with an improvement in the glycemic profile by reducing fasting blood glucose (FBG), fasting insulin, and Homeostasis Model Assessment of Insulin Resistance (HOMA-IR). In addition, nano-curcumin supplementation was shown to increase high density lipoprotein cholesterol (HDL-C). Hypolipidemic effects (reduction of TG, TC and LDL-C) of this compound have been demonstrated in patients with dyslipidemia (triglyceride [TG] > 150 mg/dl; total cholesterol [TC] > 200 mg/dl; and low-density lipoprotein cholesterol [LDL-C] > 100 mg/dl). Declines in C-reactive protein (CRP), interleukin 6 (IL-6) and systolic blood pressure (SBP) were also found, which show a beneficial anti-inflammatory and hypotensive effect of nano-curcumin supplementation (140). It is also worth mentioning that curcumin has other effects as well, such as anti-cancer properties. It is therefore a nutraceutical with great prophylactic and therapeutic potential, not only in the management of ASCVD (141).

**Figure 2:**
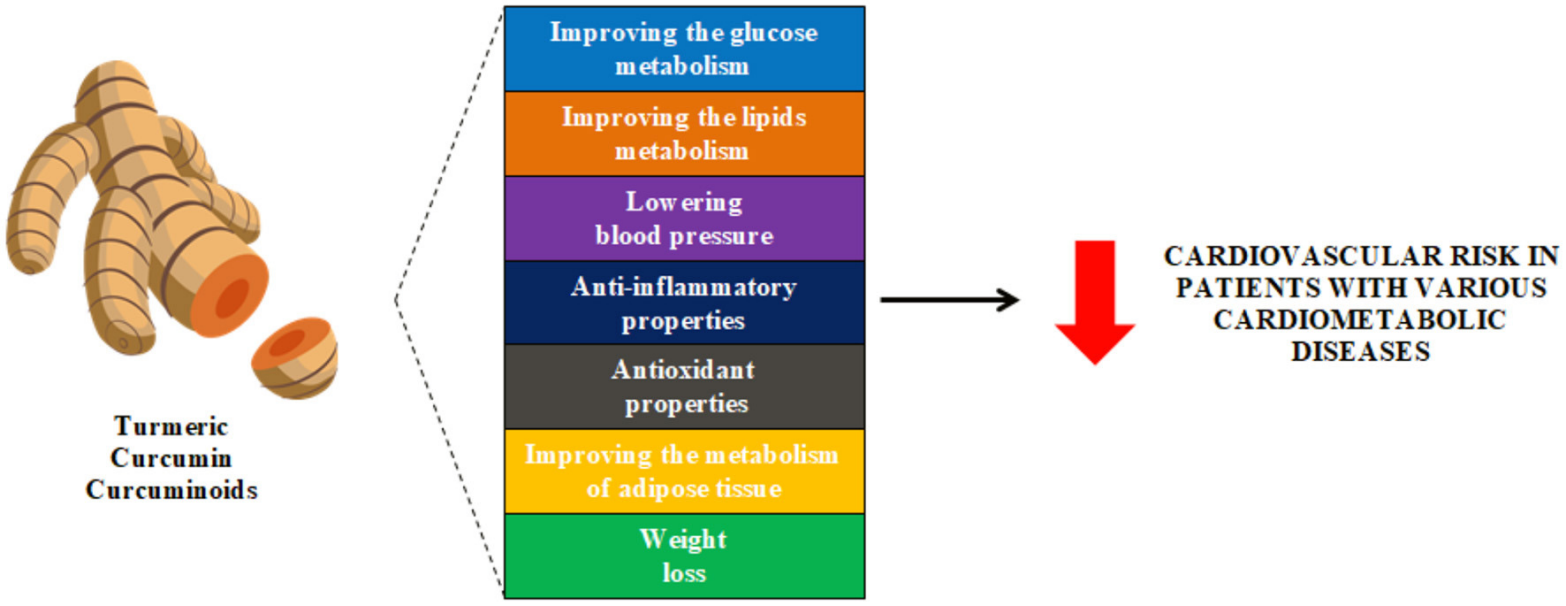
Effect of curcumin on cardiovascular risk.

Thus, curcumin has pleiotropic effects that as an adjuvant supplement can comprehensively reduce CVD risk factors and support the treatment of ASCVD in a wide variety of patient groups (Figure 2).

## Author Contributions

SS and MB contributed significantly to analysis and manuscript preparation. SS, MB, JU, AS, and PP supported valuable discussion and revised the whole manuscript. All authors collated papers, wrote the manuscript, read, and approved the final manuscript.

## Conflict of Interest

JU is CSO at Nomi Biotech Corporation. PP owns four shares in AstraZeneca PLC and has received honoraria and/or travel reimbursement for events sponsored by AKCEA, Amgen, AMRYT, Link Medical, Mylan, Napp, Sanofi. MB: speakers bureau: Amgen, Herbapol, Kogen, KRKA, Polpharma, Mylan/Viatris, Novartis, Novo-Nordisk, Sanofi-Aventis, Teva, Zentiva; consultant to Amgen, Daichii Sankyo, Esperion, Freia Pharmaceuticals, Novartis, Novo-Nordisk, Polfarmex, Sanofi-Aventis; Grants from Amgen, Mylan/Viatris, Sanofi and Valeant; CMO at Nomi Biotech Corporation. The remaining authors declare that the research was conducted in the absence of any commercial or financial relationships that could be construed as a potential conflict of interest.

## Publisher's Note

All claims expressed in this article are solely those of the authors and do not necessarily represent those of their affiliated organizations, or those of the publisher, the editors and the reviewers. Any product that may be evaluated in this article, or claim that may be made by its manufacturer, is not guaranteed or endorsed by the publisher.

## Footnotes

^1^https://pubchem.ncbi.nlm.nih.gov/compound/969516 (accessed January 29, 2022).

^2^https://www.epa.gov/oppt/exposure/pubs/episuitedl.htm (accessed January 29, 2022).

^3^https://www.efsa.europa.eu/en/efsajournal/pub/1679 (accessed January 29, 2022).
